# Bone Regeneration with Mesenchymal Stem Cells in Scaffolds: Systematic Review of Human Clinical Trials

**DOI:** 10.1007/s12015-024-10696-5

**Published:** 2024-02-26

**Authors:** Astero Maria Theodosaki, Maria Tzemi, Nikiforos Galanis, Athina Bakopoulou, Eleni Kotsiomiti, Eleni Aggelidou, Aristeidis Kritis

**Affiliations:** 1 Research Methodology in Medicine and Health Sciences, School of Medicine, Aristotle University of Thessaloniki, Thessaloniki, Greece; 2 School of Medicine, Aristotle University of Thessaloniki, Thessaloniki, Greece; 3 1st Orthopaedic Department, George Papanikolaou Hospital, Aristotle University of Thessaloniki, Thessaloniki, Greece; 4 Department of Prosthodontics, Faculty of Dentistry, Aristotle University of Thessaloniki, University Campus, Dentistry Building, 54124 Thessaloniki, Greece; 5 Department of Physiology and Pharmacology, Faculty of Medicine, Aristotle University of Thessaloniki, University Campus, 54006 Thessaloniki, Greece; 6 Regenerative Medicine Center, Basic and Translational Research Unit (BTRU) of Special Unit for Biomedical Research and Education (BRESU), Faculty of Health Sciences, School of Medicine, Aristotle University of Thessaloniki, Thessaloniki, 54636 Greece; 7 Postgraduate program of Research Methodology in Medicine and Health Sciences, Medical School, Aristotle University of Thessaloniki, Thessaloniki, Greece; 8 Thessaloniki, Greece

**Keywords:** Mesenchymal stem cell, Scaffold, bone, Systematic review, Biomaterial

## Abstract

The aim of the study is to determine the effectiveness of stem cells in scaffolds in the treatment of bone deficits, in regard of bone regeneration, safety, rehabilitation and quality of life in humans. The systematic review was conducted in accordance with PRISMA 2020. A systematic search was conducted in three search engines and two registries lastly in 29-9-2022.for studies of the last 15 years. The risk of bias was assessed with RoB-2, ROBINS- I and NIH Quality of Before-After (Pre-Post) Studies with no Control group. The certainty of the results was assessed with the GRADE assessment tool. Due to heterogeneity, the results were reported in tables, graphs and narratively. The study protocol was published in PROSPERO with registration number CRD42022359049. Of the 10,091 studies retrieved, 14 were meeting the inclusion criteria, and were qualitatively analyzed. 138 patients were treated with mesenchymal stem cells in scaffolds, showing bone healing in all cases, and even with better results than the standard care. The adverse events were mild in most cases and in accordance with the surgery received. When assessed, there was a rehabilitation of the deficit and a gain in quality of life was detected. Although the heterogeneity between the studies and the small number of patients, the administration of mesenchymal stem cells in scaffolds seems safe and effective in the regeneration of bone defects. These results pave the way for the conduction of more clinical trials, with greater number of participants, with more standardized procedures.

**Graphical Abstract:**

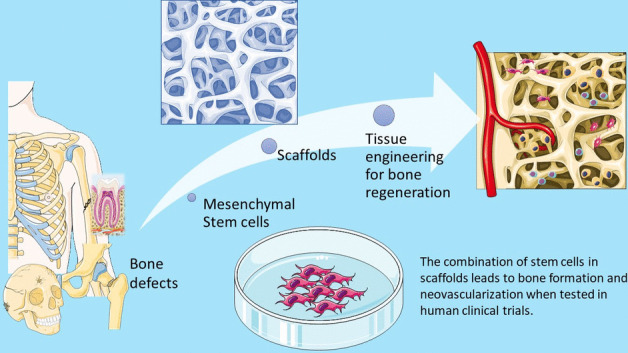

**Supplementary Information:**

The online version contains supplementary material available at 10.1007/s12015-024-10696-5.

## Introduction

The presence of nonunion fractures and enlarged bone deficits is not a rare encounter in everyday clinical practice. Most often, they are the result of bone fractures, trauma, infection, tumor treatment, some rare syndromes, even because of age. The gold standard in the treatment of those deficits is the autologous bone graft, but it does not come without consequences such as morbidity in the donor area, post operative pain and limited amount of bone [1].

The last decades, researchers are proposing the use of stem cells in the treatment of a variety of diseases with great potential. The most used in the clinical practice are the mesenchymal stem cells (MSCs). They are multipotent stem cells, they can be isolated from almost every human tissue and have the ability to differentiate in many cell types, as chondroblast, osteoblasts, adipose tissue cells. Their isolation process is rather easy, and they can be cultivated in great numbers with genomic stability and limited ethical issues [2–4].

In clinical practice, there are many cases where the term “mesenchymal stem cell” is used recklessly. The International Society of Cell Therapy has established certain criteria in order to distinguish them from other cell type. The minimum criteria are their ability to adhere to plastic during culture, their ability to differentiate into osteoblasts, chondroblasts and adipose cells, and the expression of certain cell markers (positive in CD105, CD73, CD90 and negative in CD45, CD34, CD14 or CD11b, CD79α or CD 19, HLA-DR) [5].

The therapeutic potential of mesenchymal stem cells is accommodated in the field of tissue engineering in the effort to tissue regeneration. Tissue engineering combines engineering with life sciences in order to create “biological substitutes that restore, maintain or improve the function of tissue” [6, 7]. It deployed in three main aspects, the need of stem cells, osteoinductive molecules, and osteoconductive scaffolds. The three together will provide the necessary elements and supportive environment needed for bone formation [8, 9].It is also proposed that the process should take place in a stable environment, with mechanical stability. These four parameters are the diamond concept, proposed by Giannoudis et al. [10], that is applied in the quest of bone regeneration.

Due to the advancement in the creation of scaffolds, many materials have been proposed, and the plurality of the manufacturing techniques, gave the opportunity for scaffolds with the mechanical properties and micro and macro architecture of choice [11–14]. The essential criteria for a material to be used as a scaffold is the biocompatibility and biodegradability. They display not only osteoconductive, but also osteoinductive properties, and it is also possible, with the 3D printing technology, the simultaneous printing of cells inside the scaffolds, to gain the best result [15–18]. Several materials which meet those criteria have been used such as bioceramic materials, with the best osteoinductive properties, or natural polymers which interact with the MSCs in a more physiological manner. In order to enhance both the survival and differentiation of cells, composite biomaterials have been proposed, which combine more than two different materials, increasing the degree of tissue regeneration. However, most of the concepts of stem cells and scaffolds for bone regeneration have been tested in vitro and in animal models [19–24].

In the last decade, various systematic reviews or meta-analysis have been published referring to the regeneration of bone deficits with the administration of stem cells or the application of scaffolds both in human and animal. Recently, a network meta-analysis was published, which aimed to investigate the regeneration of periodontal defects in animals, after stem cell application [25]. Its analysis included 60 studies with 5 different types of MSCs. The strongest evidence for bone regeneration was observed when applying periodontal ligament (PDLSCs), bone marrow (BMMSCs) and dental pulp stem cells (DPSCs) on scaffolds compared to single use of scaffold alone. Correlations between the use of different MSC were mainly indirect, so they have less certainty in terms of the effect they produce.

Two recent systematic reviews and meta-analyses refer to fracture healing. In the study of Kaspiris et al., they collected studies using osteoinducing substances such as growth factors, morphogenetic bone proteins (BMP-2 -7) or PRP, as well as application of MSCs. According to their study, the use of MSCs in fractures of long bones does not appear to have affected healing compared to the control group, but neither did it show adverse reactions, including ectopic osteogenesis or malignancy. However, the main research question of the study was about the application of growth factors or cellular therapy in the treatment only of non-unions of long bone fractures, of which only three studies referred to cellular therapy, with or without the addition of a scaffold [26]. Similarly, the study of Yi et al. showed encouraging results from the use of MSCs in fractures, both in animal and human studies. Although the study assesses the administration of stem cells alone, their application seems to be effective in the treatment of bone fractures [27].

Also, the concurrent application of stem cells and scaffolds has been assessed. In two systematic reviews of animal studies, positive effects on bone regeneration were observed when using MSCs in combination with scaffolds. Even more, the addition of growth factors had better results than when not applied [28, 29].

However, due to many different scaffolds and stem cells proposed, the evidence of their effectiveness is scarce. Additionally, most of the studies and systematic reviews about bone regeneration provide small evidence in human subjects. So, the aim of this systematic review is to assess the effectiveness of the use of a combination of mesenchymal stem cells and scaffold in the treatment of bone deficits in humans. Also, we assessed the safety of this treatment and its effect in function and quality of life of patients.

## Methods

### Registration

The protocol of the current systematic review was conducted in accordance with the PRISMA-P [30] and published in PROSPERO with registration number CRD42022359049.

### Eligibility Criteria

The combined application of Stem cells in scaffolds in bone defects is an effective method for bone regeneration in humans.

P (population): people with bone deficits
I (intervention): stem cells in scaffolds
C (comparator): any other therapeutic intervention not involving a combination of stem cells with scaffolding/ absence of a control group;
O (outcome): Bone regeneration
S (study type): Clinical studies in humans

#### Population/Participants

The included studies were about patients with a bone deficit or femoral fracture regardless its position. There was no restriction in age or general health issues.

#### Interventions

The studies should had at least one group where the intervention consisted of the use of stem cells in scaffolds for the treatment of the bone defect. No restriction in the type of stem cells, scaffolds, or to a certain combination of the two was applied. The cells had to be characterized as stem cells before their application in order to include the study in the systematic review. For mesenchymal stem cells, the proposed by ISCT cell markers were used for the characterization of the cells [5].

#### Comparators

The included studies could be with or without control groups in order to assess not only the efficacy, but also the safety of the intervention. The intervention in the control group could be the use of a bone graft, stem cells or scaffolds alone, or even no intervention at all.

#### Outcomes

The main outcome assessed was the healing of the bone defect. That could be assessed with clinical and radiographic measures of the recovery of the defect. If an histological analysis was presented too, it was also assessed. Because it is a rather new treatment, the systematic review aimed to ascertain the safety of the intervention, with the report of adverse events. Also, when available, we assessed measurements of the rehabilitation of function and quality of life of the patients before and after the intervention or the difference between the intervention and control group, regarding the type of defect.

#### Study Design

We included to our systematic review only clinical trials in humans, including controlled clinical trials and randomized clinical trials. We included only studies of the last 15 years for homogeneity between the studies.

#### Language

There was no restriction by language.

### Information Sources and Search Strategy

The studies were identified by searching electronic databases, such as Pubmed(MEDLINE), Cochrane (CENTRAL), Web of Sciences and the registries Clinical trials.org, WHO International Clinical Trials Registry Platform (ICTRP) (http://apps.who.int/trialsearch/). After the selection of the final studies, a citation list scanning was also conducted. The last search was conducted in 29-9-2022 and the citation list search in the 1-12-2022. The search strategy and the date of the last search are reported in Table 1.

**Table 1:** Search strategy

Databases	Search strategy	Date of last search
PubMed (MEDLINE)	((((((((bone) OR (bone tissue engineering)) OR (bone tissue engineering[MeSH Terms])) OR (bone defect)) OR (bone deficit)) OR (bone regeneration)) AND (((((((((stem cell) OR (stem cell[MeSH Terms])) OR (adult stem cell[MeSH Terms])) OR ((cell, mesenchymal stem[MeSH Terms]) OR (cells, mesenchymal stem[MeSH Terms]))) OR (MSCs)) OR (mesenchymal stem cells)) OR (mesenchymal stromal cells)) OR (cell therapy)) OR (stem cell based therapy))) AND ((((scaffold) OR (3D scaffold)) OR (tissue scaffold*[MeSH Terms])) OR (tissue scaffold))) NOT (animal)	29-9-2022
Citation search	PubMed citation	1-12-2022
Cochrane (CENTRAL)	1. stem cell 2. MSCs 3. mesenchymal stem cells 4. mesenchymal stromal cells 5. scaffold 6. 1 OR 2 OR 3 OR 4 7. 6 AND 5 ((stem cell) OR (MSCs) OR (mesenchymal stem cells) OR (mesenchymal stromal cells)) AND (scaffold) The simplification of search strategy was chosen due to same number of results as the more complicated ones.	29-9-2022
Web Of Sciences (Clarivate)	1. bone 2. stem cell 3. scaffold 4. 1 AND 2 AND 3 5. human 6. 4 AND 5 7. animal 8. 6 NOT 7 (((bone) AND (stem cell) AND (scaffold)) AND human ) NOT animal The simplification of search strategy was chosen due to same number of results as the more complicated ones.	29-9-2022
ClinicalTrials.Org	((stem cell) OR (MSCs) OR (mesenchymal stem cells) OR (mesenchymal stromal cells)) AND (scaffold)	29-9-2022
ICTRP	scaffold	29-9-2022

### Selection Process

The articles retrieved were collected in Mendeley. Then, the Rayyan [31] was used, to facilitate the screening process in first and second level. The selection process was conducted by two independent reviewers (AMT, MT), first by choosing the appropriate articles according to their title and abstract and second, when the articles passed the first screening, by full text screening, according to the inclusion criteria mentioned above. In case of discrepancies, they were resolved by consensus with a senior author (AK).

### Data Collection Process

The data of the studies selected were collected in an Excel sheet. The collection sheet form was created in advance, and calibration tests were conducted before starting the review, so any problem was resolved before the beginning of data collection. The data collection was conducted by one reviewer and a second reviewer checked the data. In case of discrepancy, it was solved by the two reviewers through discussion. Except of the main article, any supplementary items or protocol published in a study registry were checked. The data collected included demographic characteristics of the patients, the distribution of the patients in the groups, the outcomes measured, and characteristics about the methodology used by the researchers. Also, any funding information was recorded as well.

### Data Items

The following data were extracted: Name of the first author, year of publication, patient characteristics, study design, number of patients, intervention and control therapy, type of scaffold and type of stem cells, cultivation of stem cells, type of defect, adverse events, type of measurement of the healing effect (clinical, radiographic, biopsy), type of quality of life assessment, any funding source, blinding of the researchers or the assessors of the healing effect, follow up time, results of each study, statistical analysis.

For evaluation of bone regeneration, all data were collected, whether they were radiographic or histological evaluation, for safety any adverse reactions reported were recorded as well as pain evaluation, while for the restoration of function and quality of life of patients, results from questionnaires or any other evaluation by the researchers were collected. For each type of measurement of the result, all the different measurements were extracted, for each group and for each time period. For missing data, an attempt was made to find them in other sources such as in their registration in study registries. Since they were not identified, they were left blank or with most of the information that could be found.

#### Outcomes

Primary outcomes

- Healing assessment (clinical, radiographic or histological measures)
- Safety-Adverse events

Secondary outcomes

- Function- Rehabilitation
- Quality of life

### Risk of Bias in Individual Studies

The quality of the selected studies was assessed based to the Cochrane risk-of-bias tool for randomized trials (RoB 2) [32]. The assessment criteria were the randomization process, the deviations from intended interventions, missing outcome data, measurement of the outcome and the selection of the reported result. For each domain, each article was characterized as of “low”, “some concerns” or “high” risk of bias and then the overall risk of bias judgement was reached. The studies were characterized as “low”, when the study was judged to be at low risk in all domains, as “some concerns”, when the study was judged to raise some concerns in at least one domain, with none of the domains judged as high risk, and “high” risk of bias when it was judged to be at high risk in at least one domain or to have some concerns in multiple domains.

For non-randomized clinical trials ROBINS-I tool (Risk Of Bias in Non-randomized Studies - of Interventions) [33] was used, which is an extension of RoB-2, with the addition of three domains. The first one is the assessing of confounding, which is a pre- intervention prognostic factor which can predict whether a patient receives one or other intervention. In the current setting, the main confounding could be the age of patients, the size of the defect or the time being untreated, certain diseases or a therapeutic treatment which can mediate the bone healing such as the bisphosphonates [34]. The second is the selection of participants into the study and the third is the classification of intervention. The other 4 domains are similar to RoB-2. The judgement was deduced the same way as for RoB-2.

For single arm studies the NIH tool was used (Quality of Before-After (Pre-Post) Studies with no Control group of National Heart Lung and Blood Institute) [35], which is a questionnaire of 12 questions to understand the limitations or issues of bias, characterizing them as good, fair or poor.

The assessments for each study were conducted independently by two researchers (AMT, MT) and in case of discrepancies, they were solved after discussion. For the graphic visualization of the results, the *robvis* [36] tool was utilized.

### Effect Measures

For the assessment of the bone regeneration, the mean difference and the standard deviation were used, either between the two groups in the follow up time or between before and after in the single arm studies. In case of qualitative assessments, they were transformed in standardized mean difference and standard deviation. When the p value was smaller than 0.05, they were assumed as statistically significant.

### Data Synthesis

The studies were divided in regard of having or not a control group. When single arm studies had historical studies as control groups, they were categorized with the single arm studies to diminish the bias of the analysis.

Due to heterogeneity between the studies, no meta-analysis was conducted. The results of each study were reported in a table, expressing the mean difference between the intervention and control group. The table presents all the assessments of bone regeneration, in every follow up time. When the study included more than 2 groups, the extra group was characterized as control or intervention regarding the use of stem cells in scaffolds. Also, for the assessment of safety, the adverse events were collected in a qualitative manner, in a table.

The characteristics and the distribution of the interventions were depicted in charts, created in Excel.

To assess the rehabilitation and quality of life of patients, a subgroup analysis was conducted in regard of the type of defect and were presented qualitatively. No heterogeneity test was conducted due to the different study designs and the small number of studies in each type. No sensitivity analysis was conducted.

### Reporting Bias Assessment

The publication bias was assessed by the risk of bias tools, in the risk domain due to missing results for each study, and the publication bias was assessed narratively. The conduction of tests (ex. Egger’s test) or the graphical assessment with funnel plots, were thought as inappropriate due to the heterogeneity of the studies, and the assumptions made would be misleading [37].

### Certainty Assessment

In order to evaluate the quality of evidence of all outcomes, we will use the Grading of the Recommendations Assessment, Development and Evaluation (GRADE) working group methodology [38]. Their methodology assesses the quality of evidence across five domains which are the risk of bias, consistency, directness, precision and publication bias. To achieve transparency and simplicity, the GRADE system classifies the quality of evidence in one of four levels—high, moderate, low, and very low. The results were presented in a Summary of Findings Table, made online in *GRADEpro*.

## Results

### Study Selection

**Fig. 1:**
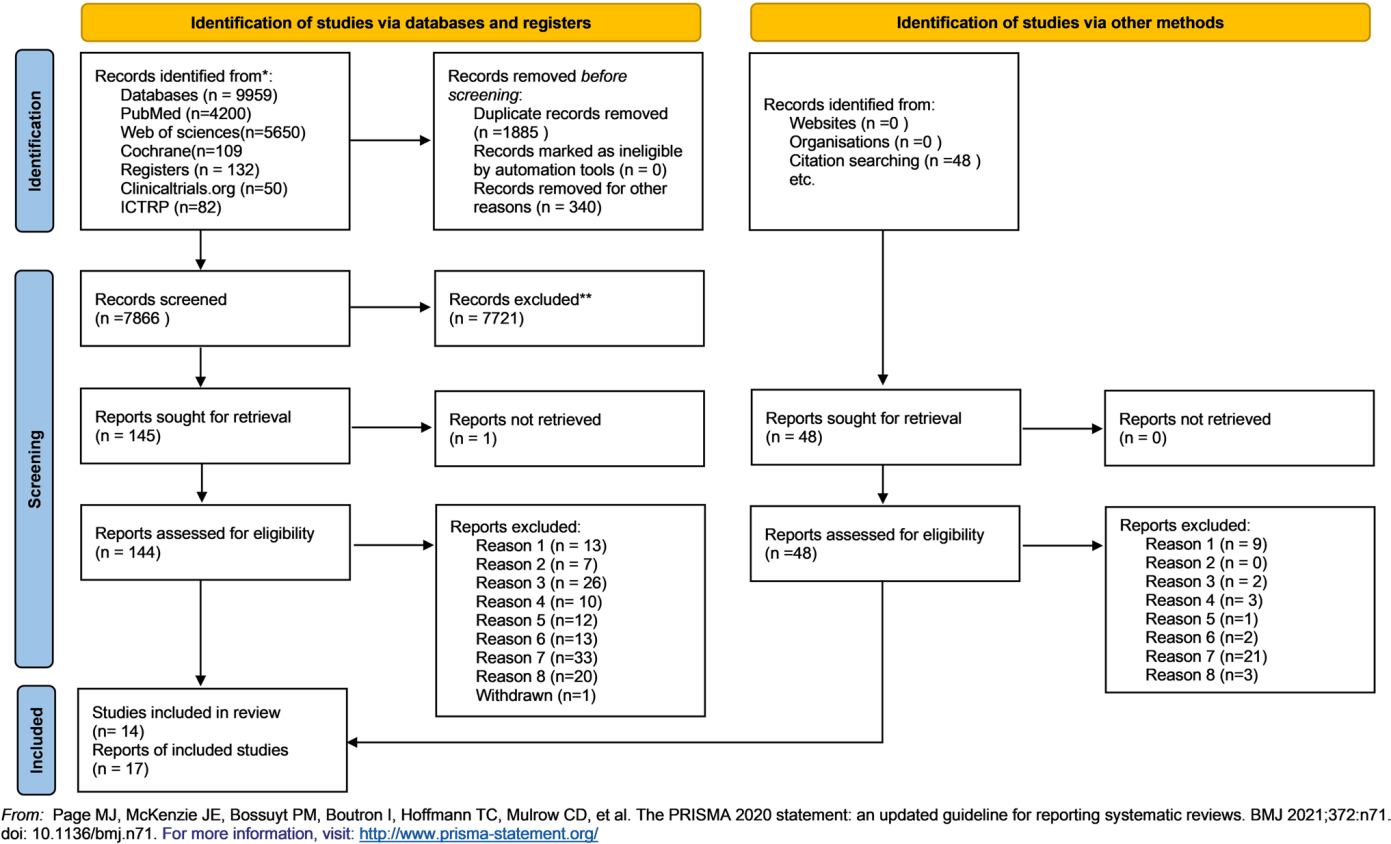
PRISMA flow chart

From the study collection process, 10,091 articles were retrieved. After the removal of the duplicates, 8206 articles arose, from which 340 were excluded because they were published before 2007, so finally 7866 articles were assessed in regard their title and then their abstract, whether they met the inclusion criteria. Of them, 145 articles were evaluated according to their full text. Also, a citation search of those 145 articles was conducted and 48 were similar to the research question and were also evaluated in full text. Finally, 14 articles were meeting the inclusion criteria. The rest of the articles were excluded because of they did not use scaffolds or stem cells in combination (No1), they were not applied in bone defects (No2), they were not applied in humans (No3), the studies were conducted before 2007 and were older than 15 years old (No 4), the studies were still in progress (No5). In addition, studies which used mesenchymal stem cells and did not report the cell markers to define the type of cells or used other markers than the certified by the ISCT, or used a combination of stem cells were excluded (No6) because of the high heterogeneity that they would cause. So, studies which did not cultivate the stem cells were also excluded, because they could not specify the cells according to their cell markers (No7). Only clinical setting trials were included, so case reports were excluded (No8). At last, the results of one study were withdrawn. The study selection process is depicted in the flow chart (Fig. 1) and the complete list of the excluded by full text studies is reported in Supplementary Data.

### Study Characteristics

The study characteristics are presented in Table 2. In the systematic review, 14 studies were included. Of them, 4 were randomized controlled studies [39–42], 5 studies were non-randomized control studies [43–47] and 5 were single arm studies [48–52], where one of them used a former clinical study as a historical control group. In the study selection process, studies with only mesenchymal stem cells were retrieved for the treatment of bone defects in humans, and no studies with embryonic or induced stem cells. In all studies, the application of stem cells in scaffolds led to bone regeneration, with minimum adverse events, mostly relevant to the surgical procedure.

**Table 2:** Study characteristics

Study	Study design	Type of defect	Number of patients	Gender Male/ Female	Age	Type of mesenchymal stem cell	Type of scaffold	Control group	Intervention group	Follow up	Adverse events	Outcomes	Conclusion
Apatzidou2021 NCT02449005 Greece	RCT	Intrabony periodontal defect	27	9 /18	20–68	a-BMMSCs alveolar Bone Marrow MSC	Collagen fleece (Parasorb®)	GB: Collagen fleece + aFPL GC: MAF	GA: Collagen fleece + aFPL + 5 × 10^6^a-BMMSCs/0,5cm^3^	6 weeks, 3,6,9,12 months 3 years	Not observed	EHI, Safety, CAL, PD, Recession, Radiographic bone fill (BF)	All variables showed significant clinical improvement with no statistical difference between the groups. Greater radiographic improvement in GA-GC/GB
Chen2016 NCT01357785 China	RCT	Intrabony periodontal defect	30 (48 intrabony defects)		18–65	PDLSCs Periodontal ligament MSCs	Bone xenograft BioOss®	Graft + GBR	Graft + PDLSCs + GBR	2 weeks, 3,6,12 months	Moderate swelling and pain in some patients	Safety, blood tests, BF CAL, PD, GR	X-ray filling of bone lesions was observed in both groups, with no statistically significant difference between the groups. The increase was proportional to time.
Hernandez- Mondaraz2020 ISRCTN12831118 Mexico	RCT	Intrabony periodontal defect	22	14 /7	59.4+-5.19 (55–64 years	DPMSCs Dental pulp MSCs	lyophilized polyvinylpyrroli-done sponge ® (clg-PVP))	Collagen sponge + collagen membrane (Biomed extend)	Collagen sponge + 5 × 10^6^ DPMSCs + collagen membrane (Biomed extend)	6 months	Pain controlled with painkillers	PD, Tooth mobility, Bone density (HU), antioxidant and interleukin levels (TAS, SOD, LPO, IL)	The increase in bone density was almost twice as high in the intervention group, with no statistically significant difference between the groups.
Khojasteh2017 NCT02859025 Iran	RCT	Alveolar cleft	10		3 adults, 7 children (8–14 years old)	BFSCs Buccal fat pad MSCs	ΝΒΒΜ (natural bovine bone mineral) Cerabone®	Iliac crest bone graft + collagen membrane	10^6^ BFSCs + 2 ml NBBM + LRCP/ Iliac crest bone graft + collagen membrane	Every 2 weeks, 6 months	There was a partial dehiscence in one patient, and partial exposure of the graft site	Soft tissue healing, volume of bone filling radiographically	An increase in newly formed bone was observed in all 3 groups, with the BFSCs + iliac bone group showing the largest increase, with no statistically significant difference.
Sanchez2019 EudraCT 2013-00435-77 Japan	Quasi RCT	Intrabony periodontal defect	20	14/6	25–70 years old	PDLSCs Periodontal ligament MSCs	Bone xenograft with collagen BioOSS - Collagen	XBS	1 × 10^7^ PDLSCs + 100 mg XBS	6,12 Months	Mild – moderate pain and swelling. Physiological closure of the lesion	PD, CAL, REC, FMPS, FMBS, Intrasurgically measured size of the lesion, quality of life questionnaire, aesthetic result assessment	An improvement in periodontal markers was observed in all 2 groups, with no statistically significant difference between the groups.
Akhlaghi2019 Iran	CCT	Alveolar bone defect	9	3/6	25,87 years (19–53)	BFSCs Buccal fat pad MSCs	HAM (human amniotic membrane)	Iliac crest bone graft + NBBM + HAM	Iliac crest bone graft + NBBM + HAM + BFSCs	5 Months	Not observed	Clinical healing, radiographic deficit filling, the feasibility of placing implants	Greater bone healing was observed vertically and horizontally in the intervention group without statistical significance
Ismail2016 NCT01626625 Indonesia	CCT	Non union of long bone fractures	10	8 /2	7–72 years	BMMSCs Bone marrow MSCs	HA- Hydroxyapatite	Iliac crest bone graft	14-18 × 10^6^ BMMSCs + HA	1–12 Months	Not observed	Assessment of pain, LEFS + DASH to assess functionality, radiographic healing of fracture with Lane-Sandee, Tiedelman	Faster healing by 3 months was observed in the intervention group, at one year the differences were assimilated between the groups.
Khojasteh2016 Iran	CCT	Alveolar bone defect	8	5 /3	38,91 years	BFPSCs Buccal fat pad MSCs	FDBA Freeze-dried bone allograft SureOss	Autologous iliac crest bone graft + FDBA	Autologous iliac crest bone graft + FDBA + 1 × 10^5^ BFPSCs	Every 2 weeks, 5 months	No inflammation of a foreign body was observed	Soft tissue healing, X-ray change in bone width, histological % of new bone	A greater increase in bone thickness was observed in the intervention group radiographically, as well as a greater percentage of new bone histologically.
Sponer2018 EudraCT2012-005599-33 Czech Republic	CCT	Femoral bone defect (hip arthroplasty)	37	15 /22	44–76	BMMSCs Bone marrow MSCs	Tricalcium phosphate β-TCP Vitoss®	(B9) Β-TCP ή (C9) sponge allograft	(A19) 15+-4.5 × 10^6^ MSC + β-TCP	6 weeks, 3,6,12 months		Hip Harris score to assess pain and function, bone healing according to Gie guidelines	Integration of the graft into the intervention group was observed at 6 months and trabecular bone formation at 12 months. There was a significant statistical difference only between group C to B.
Gjendre2018 NCT02751125 EudraCT2012-003139-50 Norway	Single arm	Alveolar bone defect	11(13), 14 sides	4 /7	65 ετών (52–75)	BMMSCs Bone marrow MSCs	Calcium phosphate biomaterial BCP (HA 20%, β-TCP 80% ) MBCP+®	-	20 × 10^6^MSCs/1cm^3^ + BCP + Regenerative membrane (PTFE)	6 Months	Not observed	Radiological bone deficit filling, histomorphometric factors of bone filling, feasibility of placement and osseointegration of implants	An increase in keratinized tissues was observed. An increase in bone was observed both in thickness and volume. Histologically, integration of BCP granules and formation of new bone were observed. Finally, the stability of the implants (Ostell values) was increased.
Gomez-Barrena2020 ORTHO-1 NCT01842477 Spain France Germany Italy	Single arm	Non union of long bone fractures	28(30)	15/13	39 +- 13	BMMSCs Bone marrow MSCs	Calcium phosphate biomaterial BCP (20% HA + 80% β-TCP) ,MBCP+®	-	20 × 10^6^ BM-hMSCs + 10 cc BCP	3,6,12 months, and intermediate reporting of adverse reactions	19 mild to moderate adverse reactions not related to the intervention were observed *	X-ray evaluation of fracture healing, Pain reduction (VAS scale)	Gradual healing of fractures was observed, where in 1 year there was complete healing in 92.8% of patients. Healing was delayed in smokers at 6 and 12 months, and to a small extent in tibia fractures. The sex and time since the initial fracture did not affect healing.
Relondo2018 NCT01389661 EudraCT2010-024246-30 Spain	Single arm	Maxillary cyst	9(11)	2 /7	36+-14	aBMMSCs Alveolar Bone marrow MSCs	3D BioMax serum Autologous cross-linked serum- scaffold matrix	-	MSCs + 3D BioMax serum	2 weeks, 3–4, 6–8 months	Not observed	Clinical assessment of healing, radiographic increase in bone density (HU)	An increase in bone density was observed in all lesions.
Takedashi2019 UMIN000007698 Japan	Single arm	Intrabony periodontal defect	12	2 /10	53,25+-9,15 ετών (43–72)	ADMSCs Adipose derived MSCs	Fibrin gel Beriplast ®	-	4,2 × 10^7^ADMPCs/mL + fibrin gel	3,6,9 months	Transient pain, poolitis and dental sensitivity, delayed wound healing	PD, CAL, BOP, GI alveolar bone growth rate, bone filling rate	There was an improvement in periodontal markers as well as the creation of a new alveolar bone, proportionally increasing over time.
Tanikawa2020 NCT01932164 Brazil	Single arm with historical control	Alveolar cleft	6	3 /3	10,16 (8–12 ετών)	DDPSCs Deciduous dental pulp MSCs	Hydroxyapatite + Collagen Sponge (Bio-Oss Collagen®)	G1: Sponge + rhBMP2 G2: Iliac crest bone graft	1 × 10^6^DDPSCs + HA and collagen sponge	1,2,3 weeks, 6,12 months	Not observed	Clinical side effects, duration of hospitalization, radiographic deficit filling, tooth eruption.	Satisfactory bone regeneration and tooth eruption (66.7%) and reduced morbidity compared to the control groups.

*RCT *Randomized clinical trial, *CCT *Controlled clinical trial(non-randomized clinical trial), *MSC *mesenchymal stem cells, *aFPL *autologous fibrin/platelet lysate, *MAF *Minimally invasive flap, EHI: Early healing index, *CAL *Clinical attachment level, *PD *pocket depth, *BF *bone fill, *REC *recession, *GBR *Guided bone regeneration, *FMPS *full-mouth plaque score, *FMBS *full-mouth bleeding score, *OHIP-14* Oral Health Impact Profile-14,*VAS* Visual analog scale, *LEFS* Lower extremity functionality scale, *DASH *Disabillities of the arms, shoulder and hand score, *LRCP *Lateral ramus cortical bone, *rhBMP2* recombinant human bone morphogenetic protein-2

#### Type of Defect

**Fig. 2:**
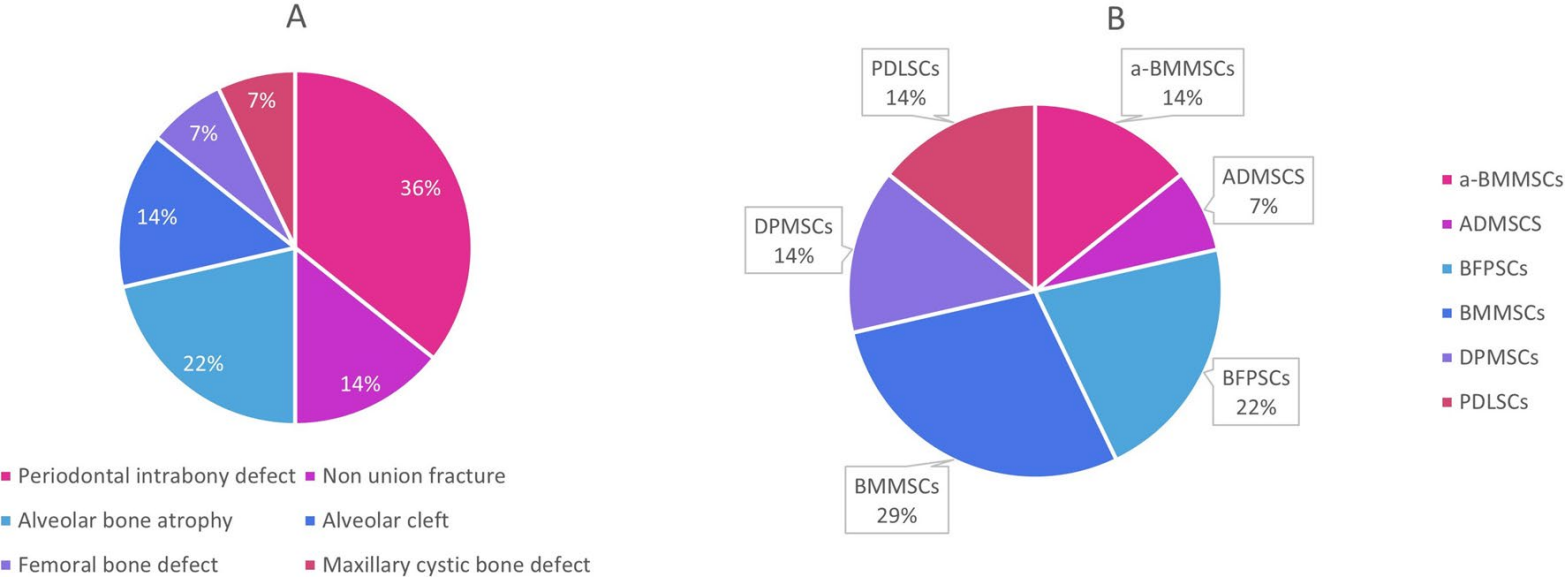
**A** Type of Defect, **B** Origin of Mesenchymal Stem Cells. (a-BMMSCs: alveolar Bone Marrow Mesenchymal Stem Cells, ADMSCs: Adipose-derived Mesenchymal Stem Cells, BFPCs: Buccal Fat Pad Stem Cells, BMMSCs: Bone Marrow Mesenchymal Stem Cells, DPMSCs: Dental Pulp Mesenchymal Stem Cells, PDLSCs: Periodontal Ligament Stem Cells)

The included studies were about 6 different type of bone defects. Specifically, 5 studies were about infrabony periodontal defects, 3 about alveolar bone atrophy, 2 about alveolar cleft, 2 about non-union in long bones and other bone defects as in the femoral bone and as a cystic bone defect of the maxilla. The distribution is depicted in the Fig. 2A.

#### Mesenchymal Stem Cells

The MSC used can be divided in three categories: MSC from bone marrow, from the iliac crest or the alveolar bone, MSC of dental origin, as from the dental pulp or the periodontal ligament, and MSC of the adipose tissues, from the buccal fat pad or abdomen. The distribution is depicted in the Fig. 2B.

#### Cultivation

**Fig. 3:**
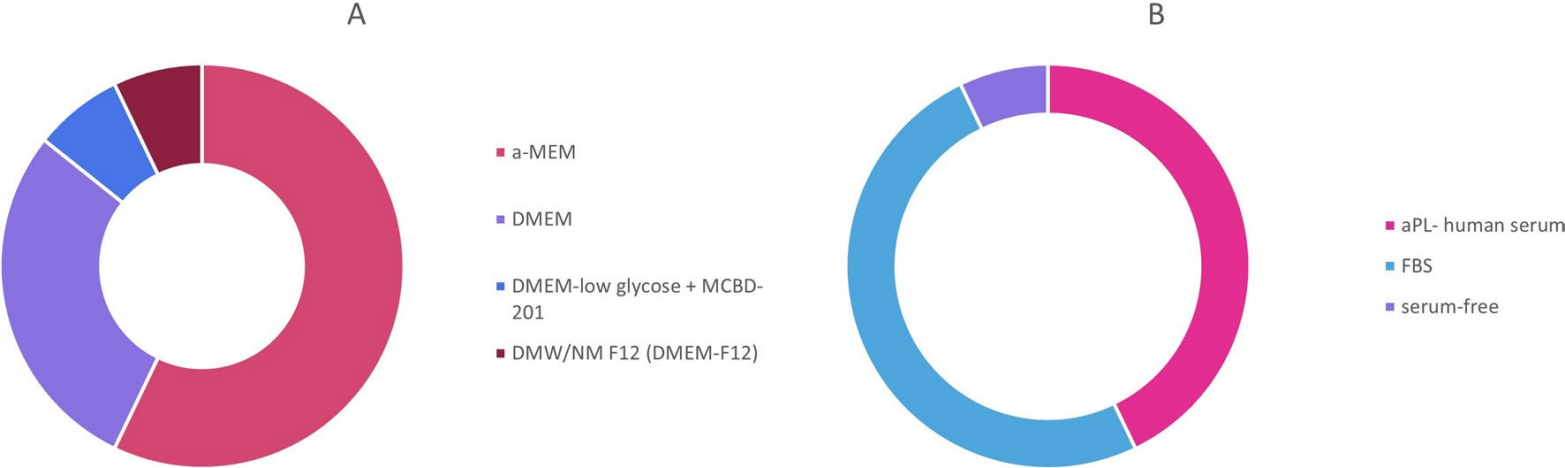
**A** Culture medium, **B** Serum

**Fig. 4:**
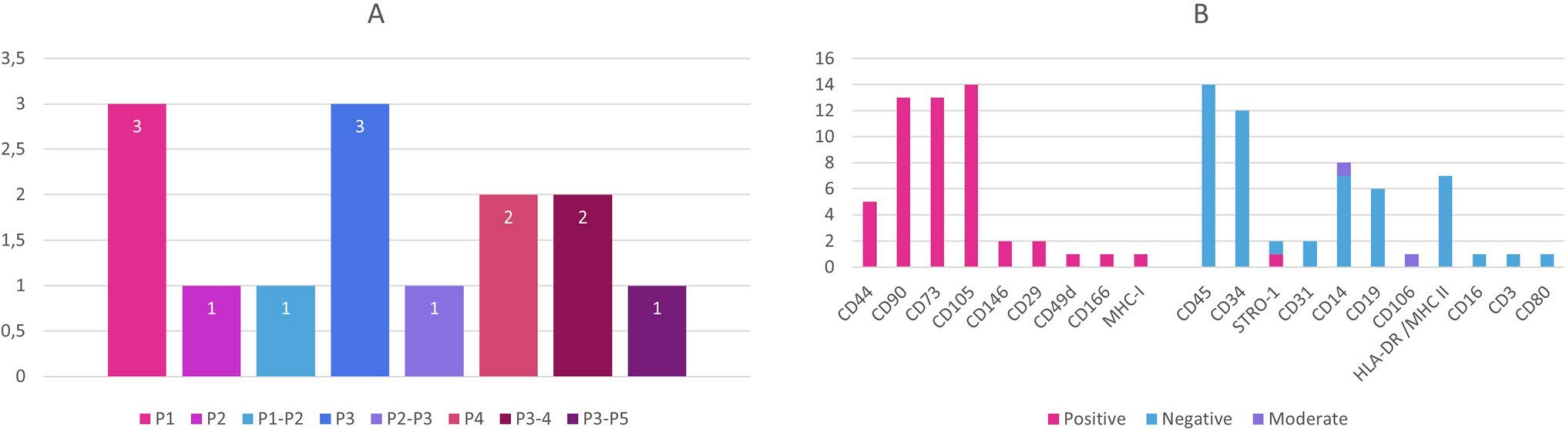
**A** Passage number, **B** Cell markers

Regarding the MSC origin, different procedures were followed. Additionally, there were differences in the cultivation medium used, the addition of serum or other additives. The number of passages did not surpass the 5 passages and all studies used 10^5^-10^7^ cells. The cultivation characteristics of the studies are presented in Table 3; Figs. 3 and 4A.

**Table 3:** Cultivation characteristics of studies

Study	Isolation technique	Enzymatic digestion	Medium	Serum	Antibiotics	Additives	Passage	Number of cells	Time of cultivation	Ex vivo seeding of MSC inside the scaffold before clinical application
Akhlaghi2019	Vestibular incision distal to the maxillary 2nd molar. 3–5 ml of buccal fat pad	Collagenase PBS for 30 min	DMEM	HS 10%			3	10^6		BFSCs were seeded over the HAM and the construct was cultured in DMEM + 10% human serum
Apatzidou2021	Alveolar bone biopsy	Enzyme mixture of 3 mg/ml collagenase-I and 4 mg/ml dispase-II (Invitrogen)	a-MEM	FBS 15% lot-selected, not heat inactivated	50 µg/ml gentamycin	100mM L-ascorbic acid	2	5 × 10^6	18–24 days	aBMMSCs were suspended in 100 µl FPL and loaded in collagen fleece
Chen2016	Extracted teeth were rinsed PB for 3 min for a total of 5 times and PDL was separated from the root	solution of 0.2% collagenase type I (Sigma) for 15 min at 37 °C	a-MEM	FBS 10%	100units/mL penicilin, 100 mg/mL streptomycin (Invitrogen) (0 passage only)		4	10^7	20 days	
Gjerde2018	Bone marrow aspiration from the posterior iliac crest, 2-4 ml with 1000IU heparin (15-20 ml/patient)		a-MEM	HPL 8% (0P)		1IU/ML heparin	1	10^7	21 days	
Gomez-Barrena2020	Bone marrow aspiration with trocar from the posterior iliac crest of 2-4 ml + 1000 IU/ml heparin		a-MEM	8% aPL			1	20 × 10^6	21 days	The cells were suspended in 5% human albumin and mixed with the scaffold
Hernandez-Monjaraz2020	Dental pulp extraction from extracted tooth	3 mg/ml collagenase type I 4 mg/ml dispase in a-MEM	a-MEM				1	5 × 10^6		
Ismail2016	Bone marrow aspirate of 40 ml from iliac crest + 5000U/ml heparin		DMEM	FBS 10%			4 patient passage 1, 1 patient passage 2	15 × 10^6	4 weeks	
Khojasteh2016	Vestibular incision distal to the maxillary 2nd molar. 3–5 ml of buccal fat pad.	3 mg/ml collagenase type I and 4 mg/ml dispase in PBS for 30 min	a-MEM	hAS 10%	1% antibiotics (Gico)		3-4P	10^5		The cell scaffolds were seeded in Osteogenic medium (Invitrogen) for 7 days
Khojasteh2017	Vestibular incision distal to the maxillary 2nd molar. 3–5 ml of buccal fat pad.	3 mg/ml collagenase type I PBS for 30 min	a-MEM	hAS 10%			3-4P	10^6		
Relondo2018	Alveolar bone biopsy		DMEM containing 4.5 g/L of glucose	FBS 10%	100 U/mL penicillin and 100 U/mL streptomycin		2–3 P	10^7		The cell seeded scaffolds were maintained in Osteogenic medium for 20–30 days
Sanchez2019	Extracted teeth were rinsed PB for 3 min for a total of 5 times and PDL was separated from the root		DMEM	FBS10%	100 U/ml penicillin, 100 µg/ml streptomycin, 50 µg/ml gentamycin,	2 mM L-glutamine	3	10^7		
Sponer2018	Bone marrow aspirate 10-12 ml		a-MEM	aPL 5%	gentamycin 10 mg/ml		3	15 × 10^6	3–4 weeks	
Takedashi2022	Liposuction of adipose tissue and washed 3 times with wash buffer	0,083% collagenase in PBS	DMEM-low glycose + MCBD-201	FBS 5%	1nM dexamethasone 60 µg/mL kanamycin	100µM L-ascorbic acid, 10 mg/L insulin-transferrin-selenium solution, 10 ng/mL epidermal growth factor	4	4,2 × 10^7	14 days	
Tanikawa2020	Dental pulp extraction from extracted deciduous tooth	Trypsin 1 mg/ml	DMW/NM F12 (DMEM-F12)	FBS 15%	2% PENICILIN + STREPTOMYCIN		3-5P	10^7		

#### Cell Markers

There are differences between the cell markers studies evaluated, with each research group reporting a different number of them. The surface markers tested were those defined by the ISCT as the minimum required, as well as some additional ones. The tested cell markers in each study are shown in Table 4 and their distribution in Fig. 4B.

**Table 4:** Cell markers identified per study

MSCs	BFP	a-BM	PDL	BM	BM	DP	BM	BFP	BFP	aBM	PDL	BM	Adipose	DP
Cell markers	Akhlaghi2019	Apatzidou2021	Chen2016	Gjerde2018	Gomez-Barrena2020	Hernandez-Mondaraz2020	Ismail2016	Khojasteh2016	Khojasteh2017	Relondo2018	Sanchez2019	Sponer2018	Takedashi2019	Tanikawa2020
CD44								+	+	+	+			+
CD90	+	+	+	+	+	+		+	+	+	+	+	+	+
CD73	+	+		+	+	+	+	+	+	+	+	+	+	+
CD105	+	+	+	+	+	+	+	+	+	+	+	+	+	+
CD146		+	+											
CD29		+	+								+			+
CD49d				+										
CD166										+				
MHC-I												+		
CD45	-	-	-	-	-	-	-	-	-	-	-	-	-	-
CD34	-	-		-		-	-	-	-	-	-	-	-	-
STRO-1		-	+											
CD31			-											-
CD14				MOD	-	-	-			-	-	-	-	
CD19				-		-	-				-	-	-	
CD106				MOD										
HLA-DR /MHC II					-	-	-			-	-	-	-	
CD16												-		
CD3												-		
CD80												-		

*MSCs *mesenchymal stem cells, *BFP *Buccal fat pad, *a-BM *Alveolar Bone marrow, *PDL *Periodontal ligament, *BM *bone marrow, *DP *dental pulp, *MOD *moderate

#### Scaffolds

**Fig. 5:**
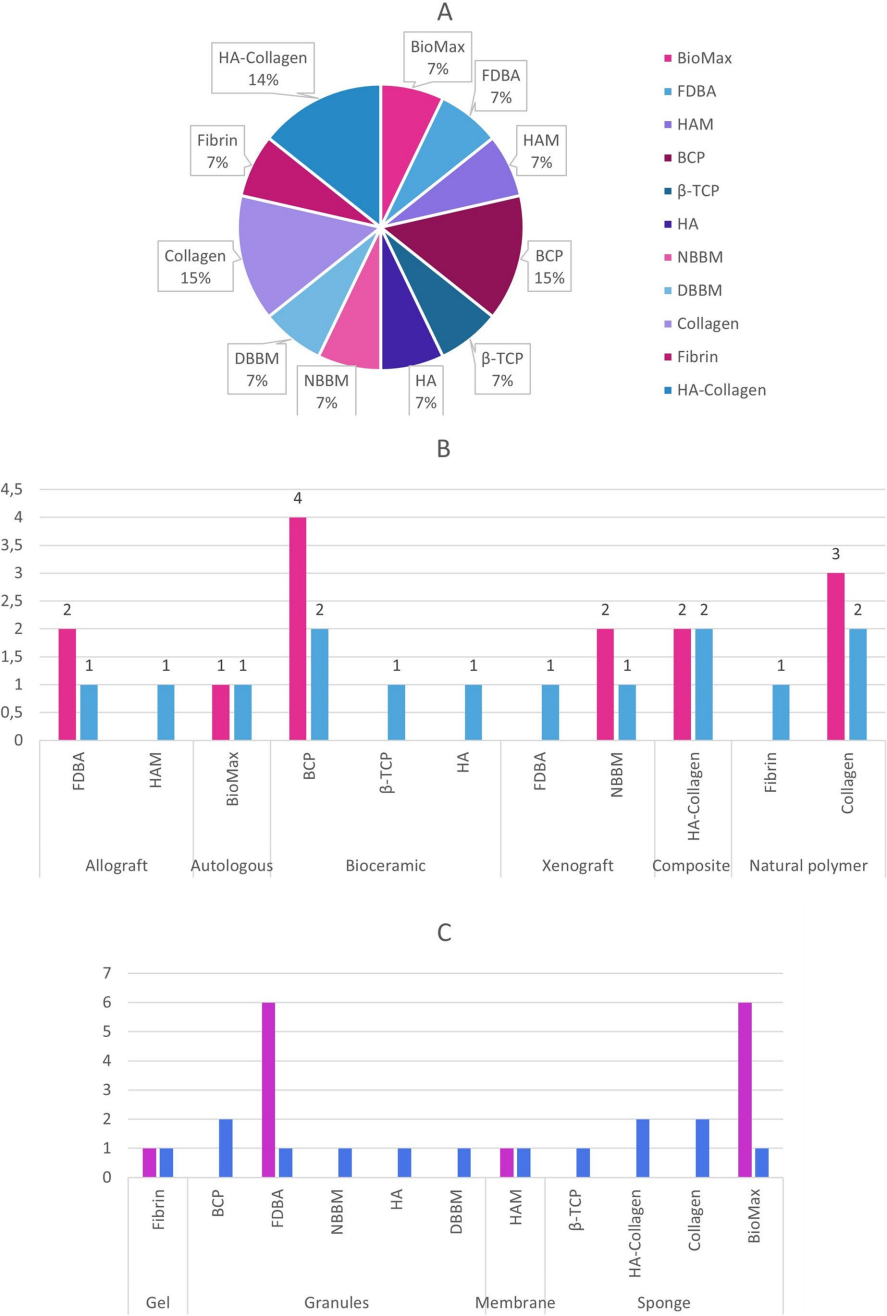
**A** Type of Scaffold, **B** Materials of Scaffolds used, **C** Composition of Scaffolds used. **B** Magenda column: Sum of each type of scaffolds, Blue column: Number of each scaffold. **C **Purple column: Sum of each type of composition, Blue column : Number of each scaffold

There was a great deal of heterogeneity in the type of scaffold used. The types of these are shown in Fig. 8. All studies used commercially standardized formulations which are used in clinical practice as graft materials, except for the study of Akhlaghi et al. [43] who used lyophilised human amniotic membrane from healthy donors, and the study of Relondo et al. [50] using an autograft of cross-linking of serum albumin-protein and glutaraldehyde (BioMax). In addition, two studies immersed the scaffold-cell complex into an osteogenic medium for 7 and 20–30 days before implantation. The characteristics of the scaffolds are shown in Fig. 5.

No correlation between the MSC origin and the scaffold was detected. However, there was a correlation between the MSC origin and the type of the defect, were the researchers usually preferred to use MSC of origin close to the type of the defect.

### Risk of Bias in Studies

Due to the different type of study design of the studies included, 3 different tools were used to assess the risk of bias.

- RoB-2

**Fig. 6:**
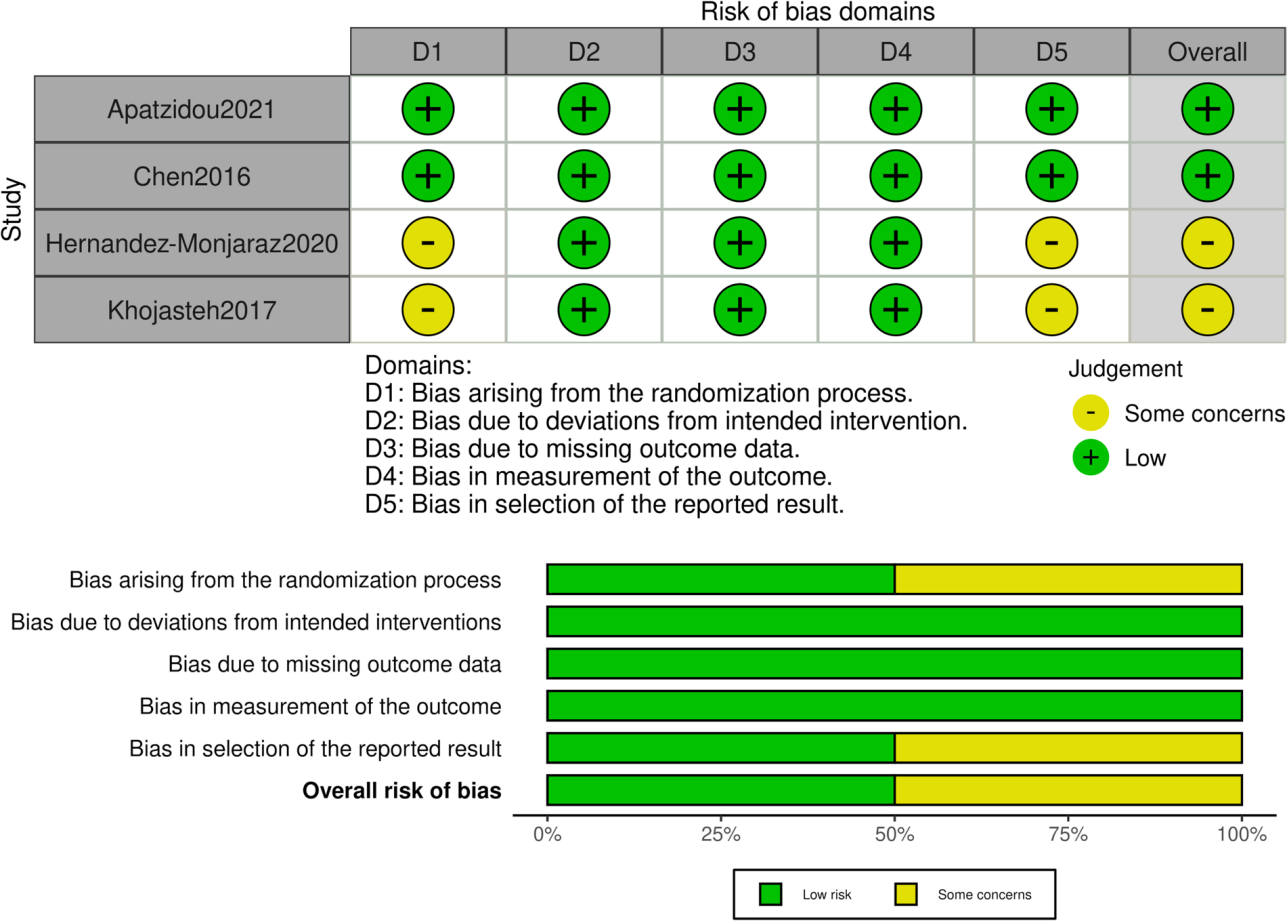
Risk of bias ROB-2 per study and per domain

Four RCT were included in the systematic review, with 2 of them showing low risk of bias and the other 2 some concerns. Specifically, about the last two, there was concern about the randomization process, which was not reported in detail, and about the selecting reporting. The results are presented in Fig. 6.

- ROBINS-I

**Fig. 7:**
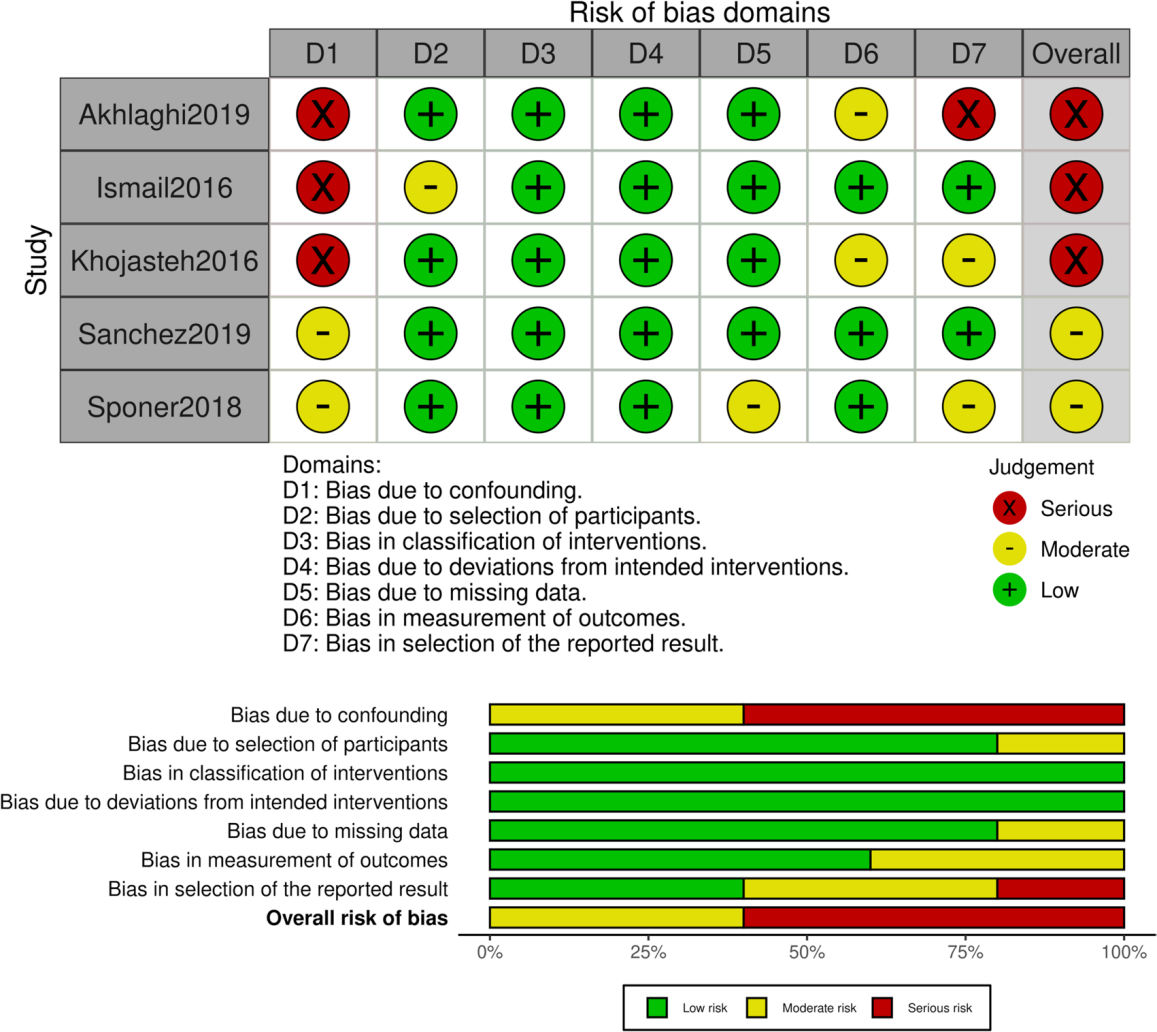
Risk of bias ROBINS-I per study and per domain

Five studies were evaluated with the ROBINS-I tool and were characterized as of medium to serious risk of bias. The main issue were the confounding factors, where the researchers did not report the main characteristics of the patients included so it was impossible to assess whether they were considered, showing serious risk, and the other 2 showed medium risk. In the domains 3 and 4, about classification of intervention and deviations from intended interventions, all studies were of low risk of bias, due to the surgical manner of the intervention. In regard of the selection of the reported result, one study was assessed with serious risk of bias, because it did not report the results of the histological assessments, which was mentioned in the “Methods” section of their report. The results are presented in Fig. 7.

- NIH

**Fig. 8:**
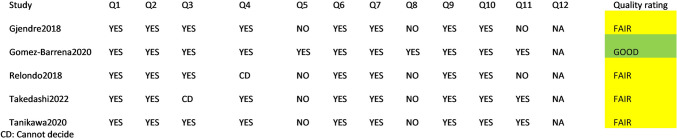
Risk of bias NIH tool

With the NIH tool, 5 studies were assessed, of which 4 were characterized as fair and 1 as good. The results are reported in the Fig. 8.

To conclude, the studies were mainly characterized with low or medium risk of bias.

### Results of Synthesis

Even though the studies investigated the same research question, they differed in their design, the defect type and the physiology of it, the risk of bias, and the assessment method of the bone regeneration. So, no metanalysis was conducted. The results of the main outcomes are presented in Tables 5 and 6.

**Table 5:**
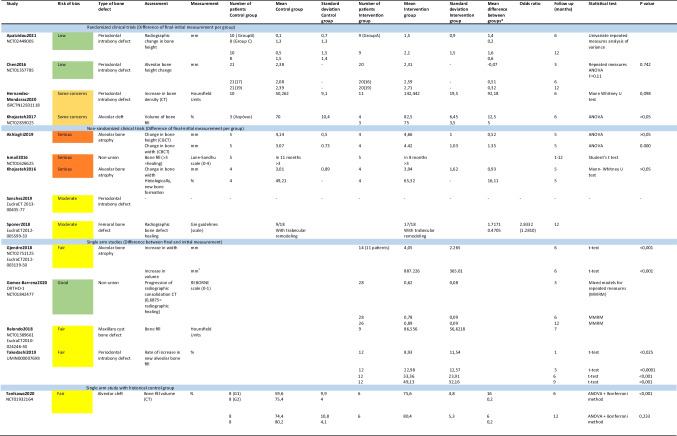
Bone regeneration

*Mean Intervention group – Mean Control Group

**Table 6:** Adverse events

Study	Control of side effects	Side effects
Apatzidou2021 NCT02449005	Allergic reactions to medication (antibiotics and chlorhexidine agents) or cultivated products, Inflammation, discomfort (pain, swelling, tenderness), injury to anatomical structures, including postoperative bleeding at the site of biopsy and/or at the site of bone defect Microbial contamination (fungi, bacteria including mycoplasma) Disturbed healing, i.e., exposure of graft materials, opening of flaps, tissue necrosis Hypothetical risk of the cells within the tissue engineering construct to undergo modification (general, phenotypic) Vitality of teeth / pathology of the pulp Integrity of oral tissues Comfort and aesthetics Unpredictable / unexpected symptoms Concomitant systemic disease or events related to quality of life	Early clinical observations revealed partially incomplete flap closure in 2 subjects of Group s-B(control), while fibrin formation along the intersection line was occasionally detected in all groups. None of the patients experienced pain in the first two weeks. All surgeries were reportedly well tolerated by patients at 12 months, while one patient (Group-B) was not satisfied with postoperative soft tissue remission.
Chen2016 NCT01357785	Screening for chromosomal changes in the karyotype Check for side effects and complications in rechecks. Blood tests before surgery, 2 weeks, 3 and 12 months after.	No patient reported any complication other than moderate swelling and pain without needing treatment. Blood tests: within normal measurements nor at the concentrations of inflammatory markers (IgA, IgG, IgM, C3, C4) 2 patients were albumin positive 1 patient was positive for glucose (control group)
Hernandez- Mondaraz2020 ISRCTN12831118	Weekly healing check-ups.	No complications were reported, beyond slight pain controlled with pain relievers
Khojasteh2017 NCT02859025	Soft tissue healing was checked every 2 weeks.	1 – partial opening in the wound - treated with good oral hygiene and solution 1 - partial exposure of the buccal cortical bone of the mandibular branch from which the graft was taken
Sanchez2019 EudraCT 2013-00435-77	Measurement of anti-inflammatory drugs taken by patients Control of wound healing using a qualitative indicator. Presence of edema. Adverse reactions with questionnaire and clinical overview of the area.	No patient reported any adverse event, other than the common side effects of regenerative periodontal surgeries. Mild-moderate swelling and pain in the first week Dentin hypersensitivity Primary wound closure in all patients More anti-inflammatories in the observation group with no statistically significant difference.
Akhlaghi2019	Soft tissue healing was checked every 2 weeks.	There was successful bone healing without a trace of inflammation, dehiscence or abscess formation and foreign body reaction
Ismail2016 NCT01626625	It was checked for one time per month for 12 months. Pain was controlled with VAS (visual analog scale)	No inflammation, immune response, malignancy or any other adverse reaction were observed. Pain was significantly reduced in all patients after 4 weeks, but more quickly in the control group. At 8 months it was reset to zero in all patients.
Khojasteh2016	Soft tissue healing was checked every 2 weeks. Histological examination was performed at 5 months for an inflammatory reaction.	No inflammation or foreign body reaction was observed
Sponer2018 EudraCT2012-005599-33	A clinical examination was performed 1 day before surgery, 6 weeks, 3,6,9,12 months after and evaluation of the pain according to the Harris hip score. All adverse reactions were recorded.	In the intervention group (not considered relevant to the intervention by the authors) 1 patient - pulmonary embolism at 10 weeks, successfully treated with thrombolysis 1 patient – dislocated at 4 months, treated with closed reduction of a correctly implanted prosthetic 1 patient – urinary tract infection at 4 months – antibiotics 1 patient – death due to pharyngeal hematoma 9 months after 1 patient – descensus uteri uterus 11 months- gynecological intervention 1 patient - post-dysplastic osteoarthritis 12 months after -contralateral primary hip arthroplasty In the control groups 1- suffered dislocation at 4 months, treated with open reduction 1 -intraoperative arrhythmia- pharmacological control 1 – femoral diaphyseal stress fracture 17 months later
Gjendre2018 NCT02751125 EudraCT2012-003139-50	According to the guidelines of the European Medicines Agency, adverse reactions were divided into local or systemic.	No postoperative infections in any of the transplants or at the donor site. 1 patient -moderate pain after augmentation and after the exposed membrane had to be removed. – others only minor pain No other adverse reactions were observed throughout the follow-up.
Gomez-Barrena2020 ORTHO-1 NCT01842477	Detection of local and general complications for 12 months of follow-up, but specifically required staggering in the first four patients by two weeks, adverse events reporting (AE) at 3,6 and 12 months, severe adverse events (SAE) and suspected unexpected and serious adverse reactions (SUSAR) at any time, and required by the regulatory framework (Eudravigillence - European Union Pharmacovigilance database; the competent authorities and to the Ethics Committees). The identification and reporting of each AE, SAE and SUSAR was done by physicians/researchers in each clinical center coordinated by an external Clinical Research Organization responsible for monitoring the recorded data in the CRF and publishing the final database. The evaluation was carried out by the consortium clinical trial research group	No AE, SAE, SUSAR were identified in relationship with the intervention. In particular, neither tumorous condition nor cell overgrowth was detected in any patient after the intervention. 19 adverse reactions were reported which were characterized as mild to moderate (fever, optalmic migraine, tonsillitis, superficial wound infection, rhinitis, sinusitis, influenza, trigeminal neuralgia, vertigo, diarrhea, breaking of locking screws lock requiring screw change, tendinitis, residual valgus knee deformity). 4 serious adverse reactions requiring hospitalization were observed. 2 patients – superficial wound inflection associated with previous surgery. 1 patient – gastric bleeding 1 patient – fatigue failure of implant – required intramedullary nail exchange
Relondo2018 NCT01389661 EudraCT2010-024246-30	Clinical control at monitoring intervals	Not reported
Takedashi2019 UMIN000007698	The oral cavity and body were checked at each visit at the monitoring intervals as well as blood and urine tests and tests. Adverse reactions were divided into Grade I, II and III according to their severity.	Mild and moderate adverse reactions were observed 12 pain within the first week in the area of surgery – 10/12 medication was required 3 discomfort in the area for over 1 week 2 increase in C-reactive protein 2 temporal inflammation of the gingival tissues − 1/2 needed treatment 1 angular stomatitis- treatment required 1 occlusion pain – treatment required 1 stomatitis 1 temporary necrosis of the gingival epithelium 1 depression of gingival tissues
Tanikawa2020 NCT01932164	Duration of hospitalization Surgical complications such as bleeding, infection, oronasal fistula,bone graft exposure or signs of ectopic bone formation were recorded.	No surgical complication was observed in the intervention group. In the control groups, in group1 37.5% of patients developed oedema postoperatively and in group 2 87.5% complained about significant donor site pain at week two. The intervention group had the shortest duration of hospitalization, together with group 1 (1 day) compared to group 2 (3 days)

#### Main Outcomes

##### Bone Regeneration

The main outcome assessed was the bone regeneration. Overall, 139 patients were treated with the used of stem cells in scaffold. In all studies, their application was successful, and the results reported were similar, or even better than the control groups, where standard care practices were used as autologous bone graft from the iliac crest or xenografts. However, the advantage of the application of stem cells in scaffold was not detected in statistically significant results in any study. That could be owed to the small sample size, because the studies were of Phase I or II. The results are presented in Table 5.

The reported adverse events are presented in Table 6. In all studies, no serious adverse events were reported, except of the study of Gomez-Barrena et al. [49, 53], where they were thought to be irrelevant to the intervention, and in the study of Sponer et al. [47], where they were due to the complication of the surgical treatment itself.

#### Secondary Outcomes

Due to the similar outcomes and characteristics of the studies regarding the defect type, it was decided to present the secondary outcomes in a subgroup manner.

##### Intrabony Periodontal Defects

There were 5 studies treating intrabony defects, 3 of them were RCTs [39–41, 46, 51]. Except bone regeneration, they also assessed the typical of periodontal health, pocket depth, clinical attachment level and gingival recession. In all studies an amelioration of the outcomes was detected but with no statistical significance, except of the study of Hernandez-Mondaraz et al. [54] (*p* < 0.001). Also, the study of Sanchez et al. [46] evaluated the oral health related quality of life and their pleasure of the aesthetic result after the end of the follow-up, with questionnaires. In all the patients an amelioration was reported, with a better advantage in the control group without statistical significance.

##### Alveolar Bone Atrophy

Three studies were about alveolar bone atrophies. An alveolar bone augmentation was conducted in order to insert dental implants for prosthetic rehabilitation. The insertion of implants was possible in all patients treated, after 4–6 months post surgically. Only the study of Gjerde et al. [48] assessed the osseointegration with Ostell measurement, the function of the dental prosthesis and the satisfaction of patients. All patients were satisfied of the result and would recommend the procedure to others. Also, the Ostell measurement increased with time.

##### Alveolar Cleft

In one of the two studies treating patients with clefts, in two adult patients they placed dental implants successfully [42]. The other study, which was a single arm study with historical control study, assessed the tooth eruption of teeth. Of the six patients, in two of them the teeth remained impacted, and an orthodontic movement was needed. In the control study, no issue with the teeth eruption was reported [52].

##### Non-Union

In the study of Ismail et al. pain was assessed with VAS questionnaires and rehabilitation with the addition of two criteria (LEFS (lower extremity functional scale) and DASH (disabilities of the arms, shoulder and hand score)) and expressed in percentage. The pain levels decreased in both groups, but sooner in the control group in the first month. The functional improvement was greater in the intervention group in the first three months (43% functional score than 27% in the control group) with statistical significance, but the difference diminished after the 7th month between the groups. In the multicenter study of Gomez-Barrena et al. [49], the pain levels during loading were assessed with a VAS questionnaire. In was assumed that when pain was lower than 30%, the stabilization of the fracture was achieved. Already in the first 3 months, there was a stabilization in the 87.5% of the patients, 88.9% of patients in 6 months, and in all patients after 12 months.

##### Other Bone Defects

In the study of Sponer et al. [47], pain and function were assessed through Harris Hip score, where an improvement was observed post-surgery in all 3 groups, with non-statistically significant difference between them.

### Risk of Reporting Bias in Synthesis

Most of the included studies had published their protocol in advance in registries, so it was possible to compare the pre-defined plan to the actual reported results. The studies were in general true to their plan, except the study of Akhlaghi and et [43]. , were, even though they report that histological assessment would be made, no results were published in their “Result” section.

Due to high heterogeneity between the studies, no funnel plot or statistical analysis were conducted. However, due to the great number of studies screened, from 5 different search engines and registries and the fact that the studies included are recent and in Phase I or II, we assume that the risk is small.

### Certainty of Evidence

Overall, the results show low certainty of evidence, except the quality-of-life which shows very low certainty. Due to the study design, and the inclusion of non-randomized clinical trials, the certainty is lowered by 1 degree. An extra degree was removed due to the imprecision of the results, due to the small sample size. In regard to the quality of life, because the results came from non-randomized and single arm trials, 2 degrees were removed. In the rest of the domains, no serious risk was detected, so no degree was removed. The results are depicted in a Summary of Findings Table (Table 7).

**Table 7:** Summary of findings table (GRADE)

Stem cells with scaffolds compared to any other treatment/no treatment for bone regeneration			
Outcomes	№ of participants (studies) Follow-up	Certainty of the evidence (GRADE)	Impact
Bone regeneration assessed with : radiographic measurement	222 (4 RCTs,4 NRCTs, 5 Single arm)	⨁⨁◯◯ Low^a,b,c,d^	The assessment of bone regeneration was done with different measurements in each studies, which made the meta-analysis imposibble. However, each study reported a result comparable or even better than the control group, but with no statistically significant result, probably due to the small study sample of the studies.
Adverse events	(4 RCTs, 5 NRCTs, 5 Single arm)	⨁⨁◯◯ Low^a,c^	All of the included studies reported whether or not were adverse events during the follow up time, which ranged from 4 to 36 months after intervention, in greater or lesser degree.
Functionality assessed with: different outcomes in regard to bone defect	(4 RCTs, 5 NRCTs, 3 Single arm)	⨁⨁◯◯ Low^a,c^	Due to the different bone defects included in the current systematic review, the fuctionality was assessed with different measures. For periodontal defects it was asssessed by periodontal assessments and mobility of teeth, for bone augmentation surgery, the sufficiency of bone growth to implantation, for alveolar cleft the impant placement of the tooth eruption, and for orthopaedic surgeries, the fuctionality with scales(exp Harris hip). All of the results showed positive rehabilitation and gain of fuctionality.
Quality of life assessed with: Quality of life and pain questionnaires	(3 NRCTs, 1 Single arm)	⨁◯◯◯ Very low^a,c^	Only a small number of studies assessed quality of life. Most studies evaluated the report of pain as an adverse event, however three orthopaedic studies assessed pain with VAS scale, with reduction of pain during follow up. One study assessed the OHRQoL and aesthetic after periodontal regeneration with positive results.
*The risk in the intervention group (and its 95% confidence interval) is based on the assumed risk in the comparison group and the **relative effect** of the intervention (and its 95% CI). **CI**: confidence interval			
**GRADE Working Group grades of evidence** **High certainty**: we are very confident that the true effect lies close to that of the estimate of the effect. **Moderate certainty**: we are moderately confident in the effect estimate: the true effect is likely to be close to the estimate of the effect, but there is a possibility that it is substantially different. **Low certainty**: our confidence in the effect estimate is limited: the true effect may be substantially different from the estimate of the effect. **Very low certainty**: we have very little confidence in the effect estimate: the true effect is likely to be substantially different from the estimate of effect.			
**Explanations** a. The outcome is assessed in a variety of study designs with a range of risk of bias from low to moderate. b. The primary outcomes of the current systematic review are bone regeneration and adverse events. Even though bone regeneration is a surrogate outcome for patients rehabilitation, it is the primary outcome to assess if the intervention was successful, so we would not downgrade for indirectness. c. Our study included studies of phase I/II, which are in general small studies assessing the safety and primary efficacy of an intervention, so we the effect size of the studies is rather small, so we downgrade for imprecision. d. There is a possible lag bias because the intervention is rather new. However, we downgraded for imprecision for that reason, we would not downgrade for publication bias, also because of the thorough search strategy not only in search engines but in registries too.			

## Discussion

The aim of this systematic review was to gather the studies using stem cells in scaffolds for bone regeneration, and to assess their therapeutic capacity, safety, impact on the restoration of functionality and quality of life of patients. Overall, in all studies, bone regeneration was successful, safe, and function was restored depending on bone defect. However, the reliability of the results is low due to the small sample of patients, so the results should be carefully interpreted. Nevertheless, this is the first evidence of their applicability to human subjects. Their application was safe, with no serious adverse events reported, the processing of stem cells was possible in a reasonable period and in most cases the discomfort of patients was similar to the other tested interventions. Moreover, the tested intervention gave even better results than the biomaterials used in everyday practice, but with no statistical significance. At last, the current systematic review highlighted the issues of heterogeneity between the different studies and promotes the standardization of the processes needed to obtain and apply those products.

The studies retrieved were of Phase I and II, whose main goal is to assess safety and plausibility of the intervention. Furthermore, the sample size of the studies was rather small to observe statistically significant difference between the control and the intervention group. However, those limitations are explained by the legislation that regulates the application of those products, usually called Advanced Therapy Medicinal Products [55]. At first, those products are tested in animal subjects before the application in human, assessing not only safety but also efficacy [56]. Therefore, there is knowledge over their capability for the treatment of the disease before the administration in humans. There are also differences in the clinical stages in human trials testing those products. In Phase I are included patients and not healthy subjects, mostly for ethical reasons, due to the peculiarity of the treatment. In Phase II the efficacy of the administration is tested, and in Phase III the safety and efficacy are validated in long term results [57]. Furthermore, in most cases, the Phases I and II are combined, due to the possibility of the incapable recruitment of the sample size desired [58]. So, due to the small period of their testing, the only available information about their efficacy comes from that type of studies. Except those issues, we encounter a great heterogeneity between the studies, including the different defect types, the origin of the MSC, the type of scaffold used and the assessment of the bone regeneration. By the GRADE assessment, the certainty of evidence was low to very low, due to the design of studies and the small sample size.

However, the current systematic review is the first, according to our knowledge, which estimates the bone regeneration after the use of stem cells in scaffolds. The eligibility criteria were strict enough to include only stem cells which were characterized as those, providing homogeneity of the cells used in the different studies. The study was in accordance with the latest guidelines to conduct a systematic review as PRISMA 2020, PRISMA – P, ROB-2, ROBINS-I and GRADE. The protocol of the study is published in advance in the PROSPERO, and any changes of it were published.

It is the first evidence of the efficacy of mesenchymal stem cells in scaffolds in bone regeneration in humans and gave prominence in the issues of clinical trials and the heterogeneity of the literature.

A great number of studies and systematic reviews have been published which investigate the effect of MSC that have been elaborated with minimally manipulation whole tissue fractions on bone regeneration. These studies differ from the studies included in this systematic review in that cells are not isolated from tissues of origin by specialized techniques but used as whole, with a mixture of cell populations, mesenchymal and non-stem cells, usually in a smaller number, while utilizing their niche to promote bone regeneration [59]. Several different protocols have been published in the literature [60–63]. Recent systematic reviews show that their application in combination with scaffolds offers improved efficiency compared to the single use of scaffolding. In addition, due to the ease of isolation and reduced cost, they may be a simple alternative [59, 64]. Thus, neither the number nor the population of cells used is clear. It is known that in tissues MSCs are found in small numbers (from 0.01% in bone marrow to 1% in adipose tissue), which is why cell culture is required for their application [63]. So, it is of paramount importance that studies using this type of product clarify these differences.

It was observed that the researchers preferred to use MSC of an origin near to the bone defect being treated. A possible indication of that, except of the knowledge of the anatomy of the area and the easier receipt from an area near to the defect, there are possible healing effects. It is known that MSC gain certain of their characteristics from the origin of their isolation. For example, the umbilical cord MSCs seem to show higher proliferation and differentiation potential than bone marrow or adipose MSC [65]. Also, MSC from different origins express different cell markers and possible differences in their immunomodulatory properties [66]. So, it is proposed that the application of MSC from an origin close to the defect site may enhance the healing of the current tissue in comparison with other MSCs [67, 68].

Apart from the origin of MSC, the cultivation process differs in the studies. First, in regard the origin of MSC, a particular procedure is applied [69]. Certain studies report that the cultivation medium or the addition of serum may influence the characteristics of MSC. For example, in a recent study, the application of a-MEM gave MSC with better osteoinductive characteristics than DMEM [70]. According to serum addition, the latest years, the human origin serums seem to prevail over the bovine serum, mostly due to ethical and economic reasons, but also because some studies indicate that the human serum is safer and promotes the cell proliferation in greater degree [71–74]. However, these are still indications and greater evidence is needed to establish that knowledge in the clinical practice.

It is well established that MSC are characterized according the ISCT criteria [5]. Most of the studies utilize those cell markers as their main criteria. Nonetheless, the studies included tested other cell markers too. As reported above, the origin of MSC can influence the expression of certain markers, but also the phase of culture or after cell differentiation [4, 70, 73, 75]. The identification of extra markers and the differences of the states noted above could ameliorate the characterization of the MSCs and may be an index of capabilities of the cells.

In addition, during the selection process, some studies came up that did not characterize their cells, resulting in ambiguity as to the type of cells they used. Thus, these studies could not be used as they would introduce bias into the review. It is clear that this heterogeneity creates confusion in the literature and possibly erroneous conclusions about the effectiveness of MSC [76].

Finally, in this systematic review it was observed that most of the scaffolds used were commercially available biomaterials which are used in clinical practice as grafts and their safety and efficacy are known. However, the literature suggests a plethora of scaffolds with composite materials and specialized manufacturing techniques such as three-dimensional printing, which after their application to bone lesions in animals and humans showed increased rates of bone healing, but without a pronounced superior biomaterial [77, 78].

According to recent bibliometric studies, the application of stem cells in scaffolds is an area of great research for the treatment of various diseases and defects [79–81]. It is clear that the advance in scaffold fabrication techniques, especially 3D printing, the combination of several materials, the simultaneous implantation of cells inside the scaffold [82], but also the knowledge for the many different stem cells that could be utilized, would provide new solutions in the current issues. Even more, there are several clinical trials in progress that estimate the effect of stem cells in scaffolds for bone regeneration (Table 8). Most of them, are randomized Phase III clinical trials, which will provide more certain evidence about the effects of the intervention in the long term.

**Table 8:** Studies in progress

Study registration number	Study design	Intervention	Control	Status	Protocol registration date
Morrison et al. 2017 10.1002/term.2459	Phase I	allogeneic mesenchymal stromal cells (MSC) on a ceramic carrier and polymer scaffold	-	In progress Primary results published	9 − 5–2017 primary results
UMIN000020398 TEOM Study	RCT Phase II	BM-MSCs, PRP, thrombin, calcium, and β-TCP	PRP, thrombin, calcium chloride and b-TCP.	In progress	15 − 1–2016 (egistered in 2019)
IRCT2015102521482N2	RCT Phase II-III	stem cells on collagen scaffold	iliac bone graft	In progress	4–9- 2016
NCT03325504 ORTHOUNION	Multicentered RCT Phase III	Autologous Cultured Mesenchymal Stem Cells + Biomaterial (High Dose): 200 × 106 cells ή 100 × 106 cells	Autologous Iliac Crest Grafting	Not yet recruiting	30–10- 2017
NCT03066245	Single arm Phase I-II	Bone marrow derived MSC seeded on biodegradable PLGA, supplemented with PL.	-	Unknown	3–5 -2018
NCT03766217	RCT Phase III	Deciduous dental pulp mesenchymal stem cells associated with hydroxyapatita/collagen	Autogenous bone will be obtained from iliac crest.	Complete – No results published	6-12-2018 13-5-2020
NCT04297813	Multicentered Phase III	Expanded, autologous mesenchymal stem cells in combination with biphasic calcium phosphate	Bone block from the ramus of the natio	Recruitinh	6–3 − 2020
IRCT20181222042074N1	Non RCT	collagen scaffold with polyglycolic acid containing mesenchymal stem cells	iliac bone grafting	In progress	13–6 -2020
ChiCTR2000036531	RCT	Peg /plga-ha particles enriched in stem cells were implanted without prp sustained release	Implanting prp-peg/plga-ha scaffold rich in stem cells, containing prp sustained release	Recruiting	24 − 8–2020
NCT03678467	Single arm	EpiBone-Craniomaxillofacial (EB-CMF) (µόσχευµα αντίστοιχο της βλάβης + ΜΒΚ)	-	Recruiting	31-3-2021
NCT04980261	RCT with triple blinding	(ORIF + FD BHA/Secretome composite)	ORIF = open reduction internal fixation + autograft	Recruiting	28-7-2021
NCT04998058	RCT with blinding	Condition medium(no stem cells) + Bone Ceramic	Bone Ceramic	Not yet recruiting	10–8 − 2022
NCT05520125	Non RCT	Mesenchymal stem cells enriched by extracellular vesicles	Standard surgical treatment of bone defects	Not yet recruiting	29–8 -2022

## Conclusion

The application of mesenchymal stem cells in scaffolds for bone regeneration is a safe intervention, with positive effects, similar to standard care, or with even better results, able to reestablish the functionality and quality of life of patients. However, the evidence of the results is low to very low, due to the small sample size and the design of studies. The following years, with the results of the studies in progress, the prosecution of bigger studies with a better design, and standardization of the processes of stem cell culture and scaffold manufacturing, will give much more evidence in the matter.

### Supplementary Information

Below is the link to the electronic supplementary material.

ESM 1 (DOCX 21.6 KB)

## Data Availability

Supplementary data are provided. Any further data or materials are available on request.
