# Supplementary material for: Bone Regeneration with Mesenchymal Stem Cells in Scaffolds: Systematic Review of Human Clinical Trials
Source: Stem Cell Rev Rep. 2024 Feb 26;20(4):938–66. doi: 10.1007/s12015-024-10696-5 (PMC11087324; doi:10.1007/s12015-024-10696-5)
Supplement: Supplementary file 1 — (DOCX 21.6 KB) [file 12015_2024_10696_MOESM1_ESM.docx]

Supplementary table 1 Search strategy

| Databases | Search strategy | Date of last search |
| --- | --- | --- |
| PubMed (MEDLINE) | ((((((((bone) OR (bone tissue engineering)) OR (bone tissue engineering[MeSH Terms])) OR (bone defect)) OR (bone deficit)) OR (bone regeneration)) AND (((((((((stem cell) OR (stem cell[MeSH Terms])) OR (adult stem cell[MeSH Terms])) OR ((cell, mesenchymal stem[MeSH Terms]) OR (cells, mesenchymal stem[MeSH Terms]))) OR (MSCs)) OR (mesenchymal stem cells)) OR (mesenchymal stromal cells)) OR (cell therapy)) OR (stem cell based therapy))) AND ((((scaffold) OR (3D scaffold)) OR (tissue scaffold*[MeSH Terms])) OR (tissue scaffold))) NOT (animal) | 29-9-2022 |
| Αναζήτηση παραπομπών επιλεγμένων άρθρων | PubMed citation | 1-12-2022 |
| Cochrane | 1. stem cell 2. MSCs 3. mesenchymal stem cells 4. mesenchymal stromal cells 5. scaffold 6. 1 OR 2 OR 3 OR 4 7. 6 AND 5   ((stem cell) OR (MSCs) OR (mesenchymal stem cells) OR (mesenchymal stromal cells)) AND (scaffold)  The simplification of search strategy was chosen due to same number of results as the more complicated ones. | 29-9-2022 |
| Web Of Sciences (Clarivate) | 1. bone 2. stem cell 3. scaffold 4. 1 AND 2 AND 3 5. human 6. 4 AND 5 7. animal 8. 6 NOT 7   (((bone) AND (stem cell) AND (scaffold)) AND human ) NOT animal  The simplification of search strategy was chosen due to same number of results as the more complicated ones. | 29-9-2022 |
| ClinicalTrials.Org | ((stem cell) OR (MSCs) OR (mesenchymal stem cells) OR (mesenchymal stromal cells)) AND (scaffold) | 29-9-2022 |
| ICTRP | scaffold | 29-9-2022 |

Supplementary table 2 Cell markers identified per study

| MSCs | BFP | a-BM | PDL | BM | BM | DP | BM | BFP | BFP | aBM | PDL | BM | Adipose | DP |
| --- | --- | --- | --- | --- | --- | --- | --- | --- | --- | --- | --- | --- | --- | --- |
| Cell markers | Akhlaghi2019 | Apatzidou2021 | Chen2016 | Gjerde2018 | Gomez-Barrena2020 | Hernandez-Mondaraz2020 | Ismail2016 | Khojasteh2016 | Khojasteh2017 | Relondo2018 | Sanchez2019 | Sponer2018 | Takedashi2019 | Tanikawa2020 |
| CD44 |  |  |  |  |  |  |  | + | + | + | + |  |  | + |
| CD90 | + | + | + | + | + | + |  | + | + | + | + | + | + | + |
| CD73 | + | + |  | + | + | + | + | + | + | + | + | + | + | + |
| CD105 | + | + | + | + | + | + | + | + | + | + | + | + | + | + |
| CD146 |  | + | + |  |  |  |  |  |  |  |  |  |  |  |
| CD29 |  | + | + |  |  |  |  |  |  |  | + |  |  | + |
| CD49d |  |  |  | + |  |  |  |  |  |  |  |  |  |  |
| CD166 |  |  |  |  |  |  |  |  |  | + |  |  |  |  |
| MHC-I |  |  |  |  |  |  |  |  |  |  |  | + |  |  |
| CD45 | - | - | - | - | - | - | - | - | - | - | - | - | - | - |
| CD34 | - | - |  | - |  | - | - | - | - | - | - | - | - | - |
| STRO-1 |  | - | + |  |  |  |  |  |  |  |  |  |  |  |
| CD31 |  |  | - |  |  |  |  |  |  |  |  |  |  | - |
| CD14 |  |  |  | MOD | - | - | - |  |  | - | - | - | - |  |
| CD19 |  |  |  | - |  | - | - |  |  |  | - | - | - |  |
| CD106 |  |  |  | MOD |  |  |  |  |  |  |  |  |  |  |
| HLA-DR /MHC II |  |  |  |  | - | - | - |  |  | - | - | - | - |  |
| CD16 |  |  |  |  |  |  |  |  |  |  |  | - |  |  |
| CD3 |  |  |  |  |  |  |  |  |  |  |  | - |  |  |
| CD80 |  |  |  |  |  |  |  |  |  |  |  | - |  |  |

*MSCs: mesenchymal stem cells, BFP: Buccal fat pad, a-BM: Alveolar Bone marrow, PDL: Periodontal ligament, BM: bone marrow, DP: dental pulp, MOD: moderate*
